# A new flea of the genus *Ctenidiosomus* (Siphonaptera, Pygiopsyllidae) from Salta Province, Argentina

**DOI:** 10.3897/zookeys.512.9713

**Published:** 2015-07-07

**Authors:** M. Fernanda López-Berrizbeitia, Michael W. Hastriter, Rubén M. Barquez, M. Mónica Díaz

**Affiliations:** 1 PCMA (Programa de Conservación de los Murciélagos de Argentina), y PIDBA (Programa de Investigaciones de Biodiversidad Argentina), Facultad de Ciencias Naturales e Instituto Miguel Lillo, Universidad Nacional de Tucumán, Miguel Lillo 205, (4000) Tucumán, Argentina; 2 Fundación Miguel Lillo; 3 Monte L. Bean Life Science Museum, Brigham Young University, 290 MLBM, PO Box 20200, Provo, UT 84602-0200, USA; 4 CONICET (Consejo Nacional de Investigaciones Científicas y Técnicas), Argentina

**Keywords:** *Ctenidiosomus austrinus*, identification key, *Phyllotis osilae*

## Abstract

A new species of flea of the genus *Ctenidiosomus* Jordan, 1931 (Siphonaptera: Pygiopsyllidae) is described from *Phyllotis osilae* J. A. Allen, 1901, from Salta Province, Argentina. This is the first time that *Ctenidiosomus* has been recorded in Argentina. A key to species of males of *Ctenidiosomus* is presented.

## Abstract (es)

Una nueva especie de pulga del género *Ctenidiosomus* Jordan, 1931 (Siphonaptera: Pygiopsyllidae) es descripta de *Phyllotis osilae* J. A. Allen, 1901, de la provincia de Salta, Argentina. Cabe mencionar que es la primera vez que el género *Ctenidiosomus* ha sido registrado en Argentina. Se ofrece una clave para los machos de las especies de *Ctenidiosomus*.

## Introduction

*Ctenidiosomus* is the only flea genus of the family Pygiopsyllidae (Pygiopsylloidea) that occurs in the Western Hemisphere and is restricted to South America. All others are found in the Australian Region. The current distribution of the genus *Ctenidiosomus* extends from Venezuela to southern Peru (Johnson 1957, Mardon 1981) and includes four species: *Ctenidiosomus perplexus* Tipton & Machado-Allison, 1972, *Ctenidiosomus rex* Johnson, 1957, *Ctenidiosomus spillmanni* Jordan, 1931, and *Ctenidiosomus traubi* Johnson, 1957. The latter species is known only in the female sex. During biodiversity studies of small mammals and birds of Northwestern Argentina, a new species of *Ctenidiosomus* was discovered and is described herein. The distribution of all species of *Ctenidiosomus* is presented on Fig. 14, and a key to known male *Ctenidiosomus* species is presented.

## Materials and methods

Biodiversity surveys of birds and small mammals were conducted in the Monte desert of Mountains and Isolated Valleys, in Salta Province, Argentina between 1998 and 2001. Ectoparasites were collected and results of those collections will be published elsewhere at a later date; however, a single male specimen representing a new species of *Ctenidiosomus* was discovered during collections in May 1999 from a male *Phyllotis osilae* J. A. Allen, 1901 and is described herein. Other mammals examined during May 1999 included 34 rodents [26 specimens of *Akodon* Meyen, 1833, four *Necromys lactens* (Thomas, 1918), and four *Phyllotis* Waterhouse, 1837] captured with Sherman live traps baited with peanut butter and oats. All rodents were subjected to a thorough post-mortem visual examination and inspected for fleas which were removed with forceps. The single flea was prepared following conventional techniques for taxonomic identification. The images were prepared using an Olympus BX61 Compound Microscope, Olympus CC12 digital camera accompanied with an Olympus Microsuite™ B3SV program in the Monte L. Bean Life Science Museum, Brigham Young University, Provo, UT, U.S.A. The landmarks used to measure the flea are described in Hastriter and Eckerlin (2003). Anatomical terms were adapted from Rothschild and Traub (1971) and the classification given by Hastriter (2012) was followed. Mammal nomenclature follows Barquez et al. (2006), Gardner (2008) and Patton et al. (2015). Unless otherwise specified, counts of setae and ctenidiae comprise only one side of flea. The host specimen was deposited in the Colección Mamíferos Lillo (CML), Universidad Nacional de Tucumán and Fundación Miguel Lillo, while the holotype of the new species was deposited in the Annexes of the CML (CML 8044).

## Taxonomy

### Siphonaptera
Pygiopsyllomorpha Medvedev, 1998 Pygiopsylloidea Medvedev, 1998 Pygiopsyllidae Wagner, 1939

#### Ctenidiosomus austrinus

Taxon classificationAnimaliaSiphonapteraPygiopsyllidae

López-Berrizbeitia, Hastriter & Díaz sp. n.

http://zoobank.org/363CEFAB-D756-4E37-AC83-F016C7331551

Figs 1–4 , 5–10 , 11–13

##### Type material.

Holotype: ♂, **Argentina**, Salta Province: ~15 km W Escoipe, on Provincial road No. 33, (25°10'25.2"S; 65°49'31.6"W), 2680m (Fig. 14), found on *Phyllotis osilae*, 17 V 1999, (CML 8044).

##### Diagnosis.

The new species can be distinguished from all species of the genus by characteristics of the distal arm of S-IX and the clasper (basimere and telomere). These include the presence of a thick sclerotization along the dorsal margin of distal arm of S-IX (Fig. 5 and 9), and by an oblique angle on the dorso-caudal apex of P1 (process) of the basimere subtended by a deep sinus (Fig. 11). General facies of the aedeagus are most closely akin to those of *Ctenidiosomus spillmanni*; however, the P1 of the basimere of the males of the three known species is rounded at the apex and the dorsal margin of distal arm of S-IX without sclerotizations (Jordan 1931, Johnson 1957, Tipton and Machado-Allison 1972), while in the new species the apex of P1 is oblique with deep sub-tending sinus and dorsal margin of distal arm with thick marginal sclerotization. Furthermore, *Ctenidiosomus austrinus* is separable from *Ctenidiosomus rex* and *Ctenidiosomus perplexus* by the lateral lobe of aedeagus not extended into narrow process, a character shared with *Ctenidiosomus spillmanni*.

##### Description.

**Figures 1–4.**
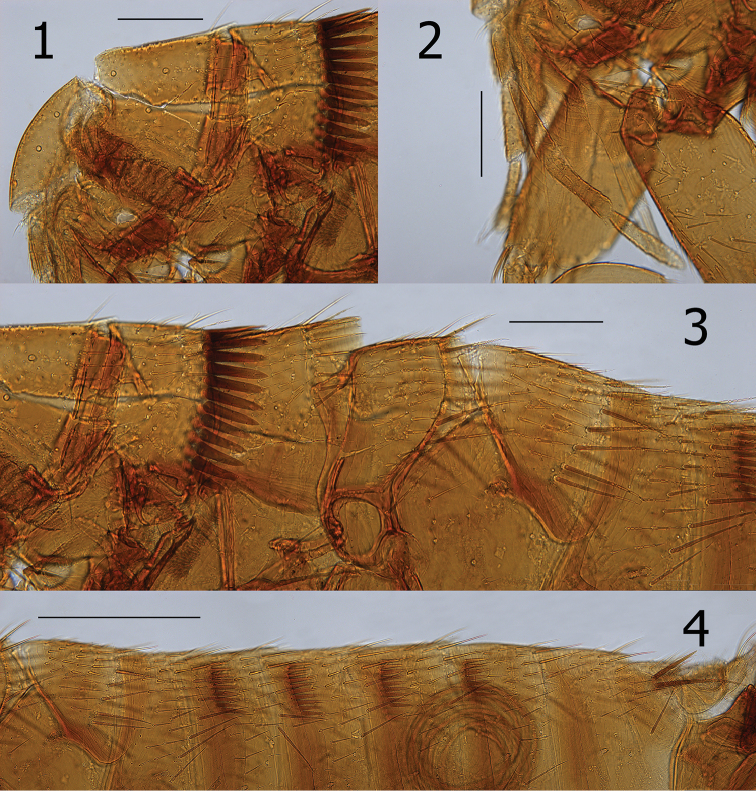
*Ctenidiosomus austrinus* sp. n., male holotype. **1** Head and pronotum **2** Labial palpus and maxilla **3** Pronotum, mesonotum and metanotum **4** Abdominal tergites. Scale: 200 µm.

Head (Figs 1–3). Frons evenly rounded, thin sclerotization throughout. Preantennal area with two placoid pits, micro-punctuations scattered over surface; two vertical rows of setae: 5–6 small setae in anterior row and three longer setae in posterior row. Eye visible, sinuate, unpigmented. Antennal scape with 27–28 small lateral setae. Pedicel with nine setae, none extending onto clavus; clavus extended beyond antennal fossa. Maxilla acutely sharp; labial palpus of four segments (excluding palp bearing maxillary segment) (Fig. 2). Post-antennal area with three placoid pits; scattered micro-punctuations over surface and several minute triangular punctiform setae between three placoid pits. Occipital area with three rows of setae; anterior row oblique with three small setae; middle row with 4–5 small setae, and main row with seven long setae plus intercalaries.

Thorax. Pronotum with comb of 23 ctenidia (both sides) preceded by three rows of setae; anterior row with three medium setae, middle with 8–9 medium setae, and main row of nine long setae plus intercalaries. Meso- and metanota with a main row of eight setae plus intercalaries (Fig. 3). Mesosternum with long seta at lower margin. Mesepimeron with three long setae. Lateral metanotal area with single long seta at posterior margin. Pleural arch and ridge well developed. Metepisternum with three long setae. Furca short and robust. Metepimeron with two rows of small setae (anterior with five, posterior with three). Rod-like abdominal link plate present as in all Pygiopsyllomorpha (Fig. 10).

**Figures 5–10.**
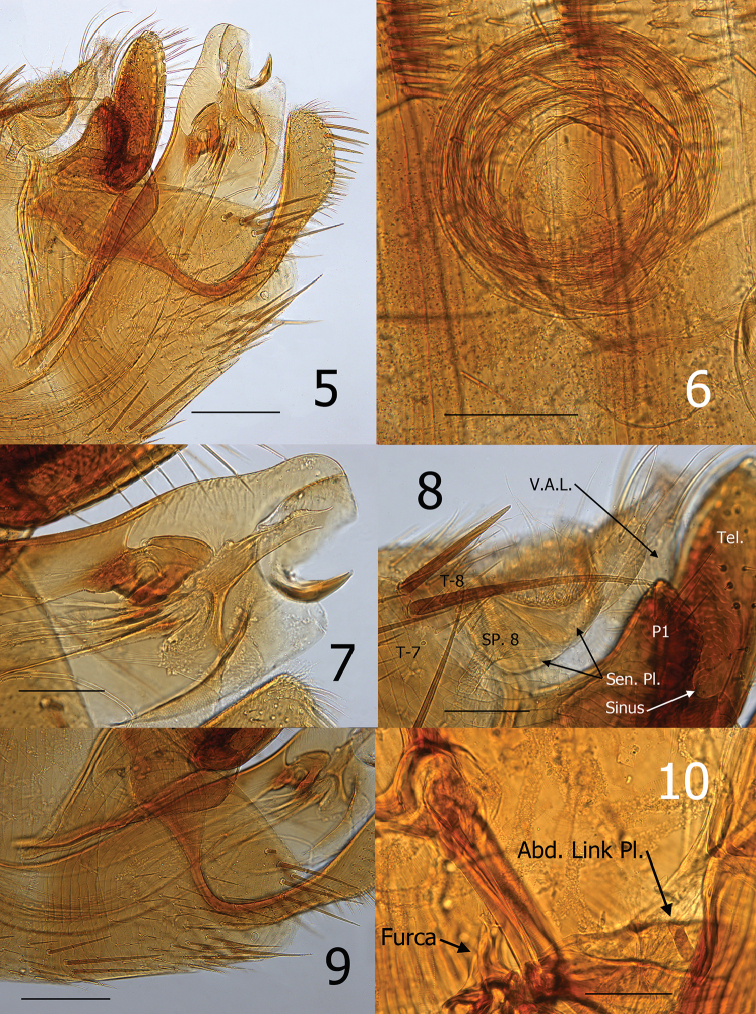
*Ctenidiosomus austrinus* sp. n., male holotype **5** Terminal segments **6** Penis rods **7** Apex of aedeagus **8** Sensilium and P1 of basimere **9** Sternum VIII **10** Abdominal link plate, furca and pleural ridge. Scale: 200 µm (**5, 6, 9**); 100 µm (**7, 8, 10**).

Legs. Fore coxa with small setae scattered over surface, one distinct horizontal row of seven setae near apex, three stout anteroapical setae. Mesocoxa and hind coxa with small setae scattered on lower outer surface. Fore femora with 4–5 minute lateral setae and one long seta at femoro–tibial joint. Meso- and hind femoro–tibial joint with one short lateral and one long mesal seta. Margin of fore, meso- and hind tibiae with seven notches. Hind tibia bearing three setae (two long, one small) in penultimate notch. Long space between notch five and six, heavily sclerotized (Fig. 12). Distotarsomere of hind leg with five lateral plantar bristles and eight preapical plantar bristles arranged in semicircle (Fig. 13).

**Figures 11–13.**
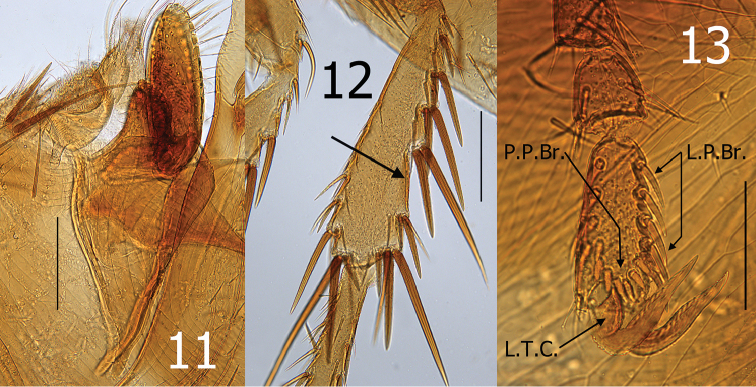
*Ctenidiosomus austrinus* sp. n., male holotype **11** Basimere and telomere **12** Hind tibia **13** Hind distotarsomere. Scale: 200 µm (**11, 12**); 100 µm (**13**).

Unmodified Abdominal Segments. Ctenidial combs on terga II–V (T-II–V). The number of ctenidia on two sides as follows: T-II 18, T-III 20, T-IV 18, T-V 15 (Fig. 4). Two antesensilial bristles (Fig. 8).

Modified Abdominal Segments. Sensilium with 18 sensilial pits. Dorsal anal lobe with three long thin setae; ventral with single long seta. (Fig. 8). Tergum VIII with seven long setae at dorso-posterior margin (Fig. 9).

Apex of P1 of basimere with oblique angle on the dorsocaudal margin with deep sub-tending sinus, manubrium with convex anterodorsal margin and apically narrowed. Telomere narrowing gradually to rounded apex. Distal arm of sternum nine (S-IX) distinctly widened apically, with five to six larger setae interspersed with smaller setae on apical margin; dorsal margin with broad marginal sclerotization (Fig. 5). Aedeagus. Median dorsal lobe rounded apically. Lateral lobe nearly parallel with upper margin of median dorsal lobe to level of sclerotized inner tube, then expands caudally into a rounded lobe that envelops inner phallosome (Fig. 7). Long thin sclerite between sclerotized inner tube and ventral margin of phallosome. Minutely membranous pouch ventral to the thin sclerite and dorsal to a thinly sclerotized ventral keel. Ford´s sclerites curved up; hyaline at base and sclerotized on apical half. Crescent sclerite with small satellite sclerite abutted against sclerotized inner tube. Sclerotized inner tube slightly narrowing towards apex; upper margin of apex longer than lower margin. Dorsal armature with minute undulations along distal half of sclerotized inner tube. Ventral armature absent. Aedeagal apodeme narrow; extending to sharp point curved upward at apex. Penis rods coiled multiple revolutions as a watch spring (Fig. 6).

Dimensions: Holotype male: 3.7 mm

##### Etymology.

The specific epithet is derived from the Latin term *austrinus* or “southern” because this new species represents the southern-most record of any known species of *Ctenidiosomus*.

##### Remarks.

The single male holotype was collected from a juvenile male specimen of the sigmodontine rodent, *Phyllotis osilae*, during the dry season in the month of May. *Ctenidiosomus perplexus* and *Ctenidiosomus rex*, were recorded on sigmodontines rodents, while *Ctenidiosomus spillmanni* was collected not only on sigmodontine rodents but also on Hystricomorpha rodents, and *Ctenidiosomus traubi* on *Caenolestes obscurus*, Order Paucituberculata (Johnson 1957, Mardon 1981) (Table 1).

**Table 1.** Hosts of the five species of *Ctenidiosomus*, actual name, and distribution are mentioned.

-	HOSTS		FLEAS
Cited as	Current Valid Name	Distribution	
Order Paucituberculata Family Caenolestidae			
*Caenolestes obscurus*§ Thomas, 1895	*Caenolestes fuliginosus* (Tomes, 1863)	Andes of Colombia, Ecuador, and northwestern Venezuela|	*Ctenidiosomus traubi*
Order Rodentia	Order Rodentia		
Suborder Myomorpha	Suborder Myomorpha		
Family Cricetidae	Family Cricetidae		
Subfamily Sigmodontinae	Subfamily Sigmodontinae		
*Akodon mollis*§ Thomas, 1894	*Akodon mollis* Thomas, 1894	Northwestern Peru to northern Ecuador, from sea level to above 4900 m¶	*Ctenidiosomus spillmanni*
*Anotomys trichotis*§ Handley, 1976	*Chibchanomys trichotis* (Thomas, 1897)	Elevation between 2400 and 2900 m in the Tachira Andes of western Venezuela and Cordillera Oriental near Bogotá, Colombia#	*Ctenidiosomus perplexus*
*Oryzomys albigularis*†‡ (Tomes, 1860)	*Nephelomys meridensis*# (Thomas, 1894)	Colombia and northern portion of Sierra de Mérida, Trujillo State, Venezuela, 1000–4000 m††	*Ctenidiosomus perplexus*; *Ctenidiosomus rex*
*Oryzomys minutus*‡ Thomas, 1917	*Microryzomys minutus* (Tomes, 1860)	Middle and high elevations in the northern and central Andes, including the Caribbean Coast Ranges and Mérida Andes of Venezuela; also in Colombia, Ecuador, Peru, and Bolivia, 800–4265 m‡‡	*Ctenidiosomus perplexus*
*Oryzomys xanthaeolus*§ Thomas, 1894	*Aegialomys xanthaeolus* (Thomas, 1894)	Coasts and mountains of southwestern Ecuador and southeastern Peru, above 2500 m††	*Ctenidiosomus spillmanni*
*Phyllotis osilae* J. A. Allen, 1901	*Phyllotis osilae* J. A. Allen, 1901	From Southern Peru to northwestern Argentina, along the eastern Altiplano and Andean slopes, 500–4000 m§§	*Ctenidiosomus austrinus* sp. n.
*Rhipidomys venustus*§ Thomas, 1900	*Rhipidomys venustus* Thomas, 1900	Endemic of Venezuela, Andes of Mérida and Trujillo, mostly above 2000 m||	*Ctenidiosomus perplexus*
*Rhipidomys*†sp.	*Rhipidomys* sp.	––––––	*Ctenidiosomus rex*
*Thomasomys cinereus*§ (Thomas, 1882)	*Thomasomys cinereus* (Thomas, 1882)	Northwestern Peru, west of the Río Marañon, 1198 to 3100 m¶¶	*Ctenidiosomus spillmanni*
*Thomasomys hylophilus*‡ Osgood, 1912	*Thomasomys hylophilus* Osgood, 1912	Cordillera Oriental, in eastern Colombia, and Cordillera de Mérida in western Venezuela¶¶	*Ctenidiosomus perplexus*
*Thomasomys laniger*‡ (Thomas, 1895)	*Thomasomys emeritus* Thomas, 1916	Venezuelan Andes in the departments of Mérida and Trujillo, 2090–3550 m¶¶	*Ctenidiosomus perplexus*
*Thomasomys* sp.†	*Thomasomys* sp.	––––––	*Ctenidiosomus rex*
*Thomasomys lugens*‡ Osgood, 1933	*Aepeomys lugens* (Thomas, 1896)||	Mérida Andes of Venezuela (Tachira and Mérida States), 1990–3500 m¶¶	*Ctenidiosomus perplexus*
Subfamily Tylomyinae	Subfamily Tylomyinae		
*Neomys* sp.§	*Tylomys mirae* Thomas 1899¶¶	Andean cordilleras of Colombia and Ecuador to the south along and through the Pacific lowlands to northwestern Ecuador, 200–1300 m##	*Ctenidiosomus spillmanni*
Suborder Hystricomorpha	Suborder Hystricomorpha		
Family Caviidae	Family Caviidae		
*Cavia* sp.§	*Cavia* sp.	––––––	*Ctenidiosomus spillmanni*

† Cited in Johnson 1957; ‡ Cited in Tipton and Machado-Allison 1972; § Cited in Mardon 1981. | According to Timm and Patterson 2008. At present, *Caenolestes obscurus* is considered synonym of *Caenolestes fuliginosus*; ¶ According to Pardiñas et al. 2015; # According to Voss 2015; †† According to Percequillo 2015a,b. Currently, *Oryzomys albigularis* is synonym of many species of the genus *Nephelomys*; we believe the cited species is *Nephelomys meridensis* because of its distribution; ‡‡ According to Carleton 2015; §§ According to Steppan and Ramirez 2015; || According to Tribe 2015; ¶¶ According to Pacheco 2015a,b, *Thomasomys emeritus* was synonym of *Thomasomys laniger*, but is currently considered a new and valid species, We determined that the cited species corresponds to *Thomasomys emeritus* by its distribution; ## According to Alvarez-Castañeda 2015, we considered the cited hosts as *Tylomys mirae* since it is the only known species of genus distributed in South America.

**Figure 14.**
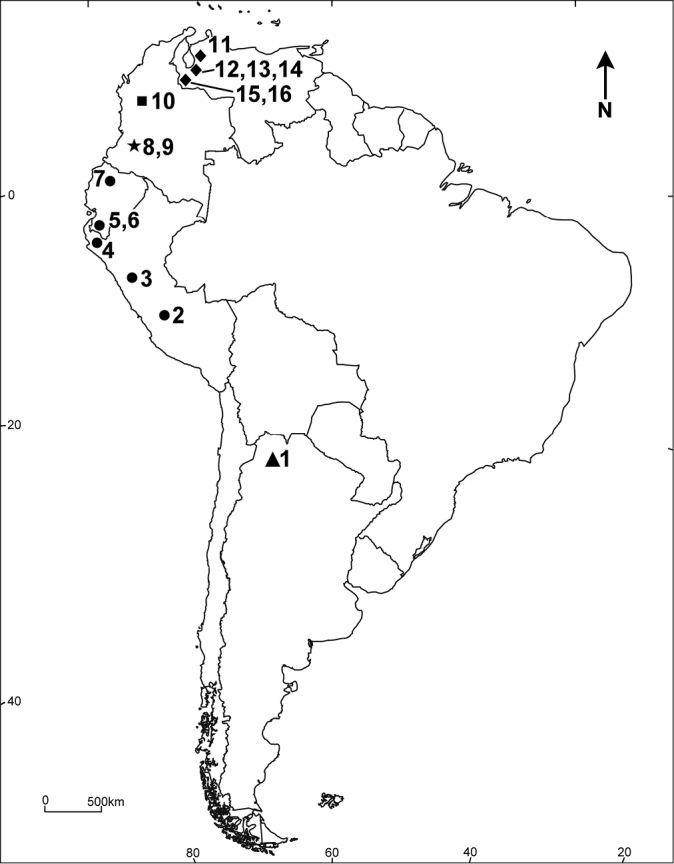
Distribution of the species of *Ctenidiosomus*. *Ctenidiosomus austrinus*: **1** Type locality: **Argentina**, Salta Province, Dept. Chicoana, app. 15 km W Escoipe, on Provincial road N°33 (25°10'25.2"S; 65°49'31.6"W), 2680 m. *Ctenidiosomus spillmanni*: **2 Peru**, Dept. Junín, Huancayo Province, El Tambo (12°01'37.88"S; 75°12'46.46"W), 3327 m; **3 Peru**, Depto. Ancasch, Huaylas Province, Huaylas (8°54'7"S; 77°50'21"W), 2067 m; **4 Peru**, Dept. Piura, Huancabamba Province, Huancabamba (5°14'22"S; 79°26'59"W), 1930 m; **5 Ecuador**, Loja Province (no specific locality) I; **6 Ecuador**, Loja Province, Alamor (4°01'14.29"S; 80°01'21.97"W), 1279 m. **7** Type locality: **Ecuador**, Pichincha Province (no specific locality). *Ctenidiosomus rex*: **8** Type locality: **Colombia**, Dept. Huila, San Agustin (1°52'39.20"N; 76°15'11.08"W), 1625 m; **9 Colombia**, Dept. Huila, San Agustin, left bank of Rio Magdalena (1°55'01.10"N; 76°20'59.06"W), 1748 m†. *Ctenidiosomus traubi*: **10** Type locality: **Colombia**, Dept. Antioquia, Sonson‡, 7 km E of Paramo (5°43'23.23"N; 75°11'11.36"W), 2049 m. *Ctenidiosomus perplexus*: **11** Type locality: **Venezuela**, Trujillo State, Trujillo Township, Trujillo, 14 km. E, near Misisi. (9°21'51.23"N; 70°18'30.89"W), 2210 m. Other distribution records: **12 Venezuela**, Merida State, near Santa Rosa (8°53'52.11"N; 70°36'55.77"W), 2040 m; **13 Venezuela**, Merida State, near Middle Refugio (8°36'07.36"N; 71°04'17.00"W), 2550 m; **14 Venezuela**, Merida State, La Coromoto (8°6'48.31"N; 71°29'54.1"W), 2500 m§. **15 Venezuela**, Tachira State, San Cristobal Township, 35 km S and 22 km W of San Cristobal, Buena Vista (7°29'43.29"N; 72°26'52.45"W), 2386 m; **16 Venezuela**, Tachira State, Buena Vista (7°29'43.29"N; 72°26'52.45"W), 2386 m; †The elevation was incorrectly cited as “2300m” in Johnson 1957; ‡The city was incorrectly cited as “Sanson” in Mardon 1981; § The elevation was incorrectly cited as “3170” in Tipton and Machado Allison 1972; I Both, the specific and unspecified localities are indicated by a point, the unspecified locality is placed in an approximate point.

The type locality of *Ctenidiosomus austrinus* corresponds to the Ecoregion “Monte desert of Mountains and Isolated valleys” (Burkart et al. 1999), where the vegetation is characterized by small and medium shrubs and cacti called “cardones” (*Trichocereus atacamensis*). Also, some scattered trees typical of the ecoregions “algarrobos” (*Prosopis alba*) are present. The soil is stony, and formed by rocks of all sizes (Burkart et al. 1999). Similar to all other species of *Ctenidiosomus*, the new species was collected at a high elevation (2680 m above sea level) (see Fig. 14 for the localities). With this report, the geographical distribution of the genus *Ctenidiosomus* is extended ~2600 km further South from its previously known southern limits of El Tambo, Huancayo Province, Department Junín, Peru (Mardon 1981). The presence of this species in Bolivia is highly probable due to its location between Peruvian and Argentine records.

There have been far fewer species of fleas described from northwestern Argentina than other regions of the country (e.g. Patagonia; see Hastriter and Sage 2009, 2011, Sanchez and Lareschi 2014), primarily due to the lack of sampling efforts. We predict that with increased surveillance in Northern Argentina and bordering countries (Bolivia, Paraguay), numerous new taxa will be discovered. Although in recent years, the sampling of small mammals has increased in Northwestern Argentina (Díaz and Barquez 2007, Ojeda et al. 2008, Ferro and Barquez 2009, Díaz and Barquez 2009, Jayat and Ortiz 2010), this region still represents one of the least studied areas in South America relative to Siphonaptera and other ectoparasites.

### Key to males of *Ctenidiosomus* species

Although females of *Ctenidiosomus austrinus* are not known, we can affirm that our specimen does not represent the currently unknown male of *Ctenidiosomus traubi* by the following characters as evidence of its distinctiveness as a new taxon when compared to the female of *Ctenidiosomus traubi*: 1) two antesensilial bristles on T-VII (three in *Ctenidiosomus traubi*), 2) presence of a distinct horizontal row of seven setae near apex of fore coxa, 3) three stout antero-apical setae on apex of fore coxa, 4) three setae (2 large, 1 small) in penultimate notch of hind tibia, and 5) pronotum with three rows of setae (two in *Ctenidiosomus traubi*).

For identification of *Ctenidiosomus* females, refer to the key by Mardon (1981).

**Table:** 

1	Ford’s sclerite of aedeagus curved down	***Ctenidiosomus rex***
–	Ford’s sclerite curved up	**2**
2	P1 of basimere rounded at apex; dorsal margin of distal arm of S-IX without sclerotizations	**3**
–	P1 of basimere oblique at apex with deep sub-tending sinus; dorsal margin of distal arm with thick marginal sclerotization	***Ctenidiosomus austrinus* sp. n.**
3	Lateral lobe of aedeagus extended into long narrow process	***Ctenidiosomus perplexus***
–	Lateral lobe not extended into narrow process, but forming a near right angle at apex	***Ctenidiosomus spillmanni***

## Supplementary Material

XML Treatment for Ctenidiosomus austrinus
